# An Integrated Fuzzy Analytic Hierarchy Process (AHP) Model for Studying Significant Factors Associated with Frequent Lane Changing

**DOI:** 10.3390/e24030367

**Published:** 2022-03-04

**Authors:** Sarbast Moslem, Danish Farooq, Arshad Jamal, Yahya Almarhabi, Meshal Almoshaogeh, Farhan Muhammad Butt, Rana Faisal Tufail

**Affiliations:** 1 Department of Transport Technology and Economics, Faculty of Transportation Engineering and Vehicle Engineering, Budapest University of Technology and Economics, Műegyetem rkp. 3, 1111 Budapest, Hungary; sarbastmoslem@hotmail.com; 2 Department of Civil Engineering, Comsats University Islamabad, Wah Campus, Islamabad 45550, Pakistan; danish.farooq@ciitwah.edu.pk (D.F.); faisal.tufail@ciitwah.edu.pk (R.F.T.); 3 Interdisciplinary Research Center of Smart Mobility and Logistics (IRC-SML), King Fahd University of Petroleum & Minerals, KFUPM, Dhahran 31261, Saudi Arabia; 4 Center of Excellence in Trauma and Accidents, King Abdulaziz University, Jeddah 21589, Saudi Arabia; 5 Department of Surgery, Faculty of Medicine, King Abdulaziz University, Jeddah 21589, Saudi Arabia; 6 Department of Civil Engineering, College of Engineering, Qassim University, Buraydah 51452, Saudi Arabia; m.moshaogeh@qu.edu.sa; 7 Development Services, Lee County Government, Fort Myers, FL 33902, USA; FButt@Leegov.com

**Keywords:** frequent lane changing, highway safety, lane change factorial model, fuzzy analytic hierarchy process, multicriteria decision making

## Abstract

Frequent lane changes cause serious traffic safety concerns, which involve fatalities and serious injuries. This phenomenon is affected by several significant factors related to road safety. The detection and classification of significant factors affecting lane changing could help reduce frequent lane changing risk. The principal objective of this research is to estimate and prioritize the nominated crucial criteria and sub-criteria based on participants’ answers on a designated questionnaire survey. In doing so, this paper constructs a hierarchical lane-change model based on the concept of the analytic hierarchy process (AHP) with two levels of the most concerning attributes. Accordingly, the fuzzy analytic hierarchy process (FAHP) procedure was applied utilizing fuzzy scale to evaluate precisely the most influential factors affecting lane changing, which will decrease uncertainty in the evaluation process. Based on the final measured weights for level 1, FAHP model estimation results revealed that the most influential variable affecting lane-changing is ‘traffic characteristics’. In contrast, compared to other specified factors, ‘light conditions’ was found to be the least critical factor related to driver lane-change maneuvers. For level 2, the FAHP model results showed ‘traffic volume’ as the most critical factor influencing the lane changes operations, followed by ‘speed’. The objectivity of the model was supported by sensitivity analyses that examined a range for weights’ values and those corresponding to alternative values. Based on the evaluated results, stakeholders can determine strategic policy by considering and placing more emphasis on the highlighted risk factors associated with lane changing to improve road safety. In conclusion, the finding provides the usefulness of the fuzzy analytic hierarchy process to review lane-changing risks for road safety.

## 1. Introduction

Each year, over 1.35 million people die, and as many as 50 million become injured in traffic accidents worldwide [1]. In 2010, the European Union repeated its intention to develop road safety by placing a mark of decreasing road fatalities by 50% by 2020, followed by a prior mark agreed in 2001 to halve the number of road fatalities by 2010. A new aim was declared by the European Commission on 17 May 2018 to halve road fatalities. The earliest set target to halve the number of severe road traffic injuries is by 2030 compared to 2020 stages [2]. According to the latest available data [3], Hungary recorded an overall rise in the number of road deaths in 2018. Accordingly, 633 persons lost their lives in traffic accidents. This represents an increasing trend in road fatalities as compared to recent years’ traffic crash data. Amongst them, human-related factors are considered to be the leading source of traffic fatalities worldwide [4]. 

The situation investigation of the Road Safety Action Program (RSAP) has revealed that human factors contribute to a high number of road crashes; therefore, solving them aids the most desired objective of highway safety programs [5]. The frequent lane-changing act is one of the human-related issues that pose a negative impact on the traffic system’s efficiency under enhanced traffic demand [6]. An investigative study [7] reported that lane changing action is one of the severe common causes of collisions in the United States. Moreover, official data records showed that at least one-third of all traffic collisions ensue as vehicles perform lane change operations or during exiting the road. In addition, recent crash data (2010 to 2017) in Middle East countries indicate that abrupt lane changes are one of the primary sources (17%) of severe traffic crashes followed by speeding (12.8%) [8].

In current years, most researchers analyzed lane-changing occurrences by applying different dynamic and statistical systems, while few studies considered game theory to observe lane-changing actions [9,10,11,12]. Moreover, the latest advancements in Artificial Intelligence (AI) have also led to promising opportunities for examining drivers’ behavior during frequent lane-change maneuvers, thereby providing key indicators for safety. Accordingly, the neural network model can evaluate lane-changing behavior in a more defined manner as compared to the multinomial logit model [13]. Li et al. (2016) [14] utilized probabilistic methods to investigate lane-changing behavior. The authors developed an exponential probability model to investigate the association among relative velocity and lane-changing likelihood relative vehicle gaps. In addition, the latest research employed a simulation-based model to estimate the effect of important elements on lane-changing [15]. Farooq et al. [16], using the designed calibrated standards for driving logic ‘conscious’ in VISSIM, examined the influence of critical traffic parameters on traffic safety during frequent lane changes. While in another recent research study [17], the authors introduced a novel procedure for validating lane-changing in the context of urban driving conditions. The human inducements from different perspectives as route change incentive, speed incentive, courtesy incentive, comfort incentive, etc., were described comprehensively using a decision-theoretical framework known as Multi-Criteria Decision Making (MCDM). The approach of grouping was considered explicitly according to the type of driving style, which can vary across different individual and driving conditions. Consequently, a decision selection procedure for lane-change operations was proposed. The proposed model yielded a more complex and frequent lane-changing behavior with various inclinations of incentives [17]. 

In recent years, fuzzy sets have been gaining widespread recognition in the application of multicriteria systems [18]. Presently, various studies merged AHP methodologies with the system of fuzzy logic that provides risk prioritization according to their threat level and produces a reliable model assessment of risk. The application of such risk assessment models can be found in various fields, such as the risk assessment of floor water incursion in coal mines [19], driver behavior criteria [20], and information technology projects [21]. Moreover, the state of urban transport supply value in Mersin was estimated by employing fuzzy-AHP methodology to create a more practical assessment method [22]. Some previous studies applied the FAHP method in road transport projects, as described in Table 1.

In current crash data, it was mainly observed that frequent lane changing is caused due to the involvement of at-fault drivers (at the crash-prone spots/locations) without highlighting the reasons following such actions [8,17,30]. Previous study findings mainly focussed on critical driver behavior factors that could influence traffic safety by utilizing statistical methods [31,32] and MCDM procedures [20,25,33], but these studies lack research specifically about the decision on highly significant main factors and subfactors designed in the AHP hierarchical model that can be involved in frequent lane changes based on experts responses using a robust FAHP model. The applied model is more efficient than pure AHP, because fuzzy sets mitigate the uncertainty of nonexpert evaluations. To analyze the important road safety issue (frequent lane changing) comprehensively, the present study has considered the well-acknowledged lane change model designed on the AHP framework [34]. In this study, the adopted model aims to inspect and prioritize the most crucial factors and subfactors affecting frequent lane change based on evaluators’ answers in order to mitigate uncertainty in nonexpert evaluations. By applying the integrated FAHP approach, the experts in the field intended to handle the vagueness of human behavior by using linguistic terms for the assessment of efficient transport systems. Currently, several studies combine AHP approaches with the system of fuzzy logic that offers risk prioritization according to their risk level and produces a consistent model for risk assessment. The use of such risk assessment models was utilized in several areas such as the risk assessment of floor water incursion in coal mines [19], information technology developments [21], and assessment of means in hazardous substance transportation [26].

This study work is designed as follows. The questionnaire survey design based on the fuzzy-AHP approach is described in Section 1. Subsequently, the case study considering a real-life subject with the arrangement of the proposed model is presented. Furthermore, it also includes the significance of main factors and subfactors affecting frequent lane changes associated with highway safety. Then, in the next section, the methodological procedures and features of the fuzzy-AHP method are presented. Afterward, the results section highlighted the most significant lane-change factors based on measured weights. After that, sensitivity analysis was employed to verify the robustness of the study results. Finally, conclusions are drawn along with some future suggestions to the experts of the specified field with statements for advanced studies.

## 2. Methodology

### 2.1. Questionnaire Survey

This study is designed to highlight the critical factor affecting frequent lane changes using the fuzzy-AHP technique. To assess road safety conditions, important data sources include roadside surveys, questionnaire surveys, and accident statistics. The questionnaire survey, in particular, if they are directed online, is a comparatively inexpensive approach to obtain indicators on road users’ behaviour and safety culture, but they rely on self-declared information. A key benefit of questionnaire surveys is that they can deliver understanding into the socio-cognitive factors of behavior, such as perceived social norm, attitudes, existing habits, or risk perception [35]. To evaluate human actions involved in road safety issues and to propose safety measures, there has been substantial research performed based on questionnaire-related studies [5,20,31,32]. DBQ was originally introduced to identify the deviant driving behavior in related earlier studies in the 1990s [36,37]. In this regard, the Driver Behavior Questionnaire (DBQ) extends out due to its dominance and longevity use among the numerous implementations [37]. The present research work is aimed at performing a questionnaire-based survey using a fuzzy scale to assess the influence of major factors on lane changing. The city of Budapest (Hungary) was considered as a case study. Accordingly, the questionnaire survey was circulated among hundred evaluators (drivers having a valid driving license) from the Department of Transport Technology and Economics at ‘Budapest University of Technology and Economics’. The response rate was 70%. Solomon (2006) [38] emphasized in his phenomenon ‘Wisdom of crowds’ that 20 participants can contribute an extreme judgment. The online survey was supported with the application of an online google form. The acquired response data of seventy participants based on the FAHP framework simulated their knowledge related to driving behavior in an efficient manner. Furthermore, the questionnaire was divided into two portions to collect relevant information. The first portion consists of essential data related to evaluators such as gender, age, education, and duration of driving license, as presented in Table 2, while the second part intended to estimate the effect of critical factors on lane-changing designed on a fuzzy scale, as reflected in the results section. As an example, Table 3 presents the questionnaire survey designed on a fuzzy scale for level 1, while the detailed questionnaire forms used for the study are provided in Appendix A. 

### 2.2. Factors Affecting Lane Changing

**Figure 1:**
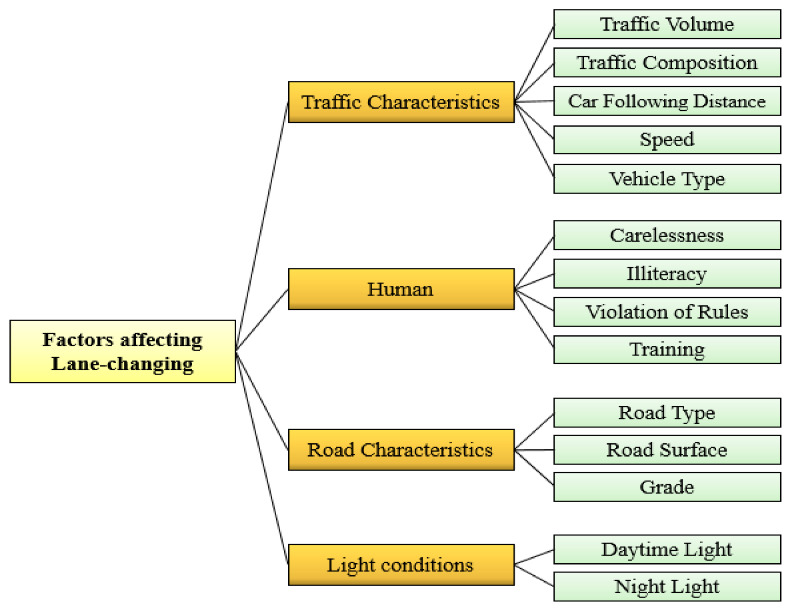
Lane change factorial model [34].

The trend of lane-changing was stated as ‘a driving practice in which along the same direction of travel the vehicle’s movement are changed between the adjacent lanes’ [39]. This work considered the crucial factors and their surrogate factors influencing lane changing and is designed on a two-level hierarchical arrangement for evaluation purposes. The first level contains four basic elements affecting traffic safety such as as ‘human attributes’, ‘traffic characteristics’, ‘road features’, and ‘lighting conditions’. For level 2, these basic elements were subsequently distributed into surrogate elements, as presented in Figure 1. A summary showing the significance of specified factors affecting lane changing is depicted in Table 4, with abbreviations and associated references.

### 2.3. Analytic Hierarchy Process (AHP)

One of the highly generally utilized techniques of MCDM analysis is the application of an Analytic Hierarchy Process (AHP), which was first introduced by Karayalcin in 1982 [56]. AHP presents an idyllic stage for complex decision-making issues. The AHP method utilizes specific mathematical procedures for handling subjective choices of an individual or a group of individuals on the sets of related criteria, evaluating and analyzing results. In many situations, the individuals are specialists in a specific area of work [57]. AHP is one of the best extensively used MCDM techniques in business, management science, and engineering. AHP aids the decision-maker in assessing complex problems with multiple conflicting and subjective measures [58,59]. In the analysis of risk, the AHP method is updated, and it can be sequenced into three major stages: (1) the formation of a hierarchical model of risk components; (2) measurement of the relative weights of risk components; and, finally, (3) the quantifiable evaluation of the risk intensity [60]. The AHP method involves the following steps:

Step#01: Designing the hierarchical model of assessment elements;
Step#02: Create the survey using a fuzzy scale (1–9) for pairwise comparisons in the hierarchical structure;
Step#03: Analyzing the consistency of pairwise comparisons;
Step#04: Estimating the aggregated weight scores;
Step#05: Developing the weight vectors and measuring the final weight scores by observing branch networks.
Step#06: Conducting Sensitivity investigation

In AHP consideration, several mutual systems were observed [58]. Sometimes, the experts in the survey do not provide a numerical decision. Alternatively, a relative verbal conception, which is highly distinctive in our daily lives, is adequate under such circumstances [61]. Nevertheless, the qualitative 1 to 9 central scale introduced by Saaty, T.L. (1980) [56] is normally utilized to estimate the elements by performing pairwise comparisons. Directed comparisons are documented by a positive reciprocal matrix, as presented in Equation (1):

(1) $$X=\left[\begin{array}{cccc} x_{ij} & x_{12} & \ldots & x_{1n}\\ x_{21} & 1 & \ldots & \vdots \\ \vdots & \ldots & 1 & \vdots \\ x_{n1} & \ldots & \ldots & 1 \end{array}\right]$$

where $x_{ij}$ denotes the factorial comparison between *i* and *j*, and *n* indicates the measurement of the pairwise comparison matrix. It also shows the number of weighed elements in the matrix and the total comparisons ($n\left(n-1\right)/2)$.

If the matrix is completely consistent, then $x_{ij}=x_{in}.x_{nj}$. For checking matrix consistency, Saaty et al. [56] determined the Consistency Index (*CI*), and it is relevant to the maximum eigen value $\left(\lambda_{max}\right).$

(2) $$CI=\frac{\lambda_{max}-n}{n-1}$$

The consistency ratio (*CR*) is subsequently determined by the following relation:

(3) CR=CI/RI

where *RI* represents the average random index based on matrix size and can be determined using the information presented in Table 5.

The matrix consistency is perfectly reliable and suitable when *CR* is less than 10%; if not, the assessor has to re-estimate the pairwise comparison matrix.

### 2.4. Triangular Fuzzy Sets

**Figure 2:**
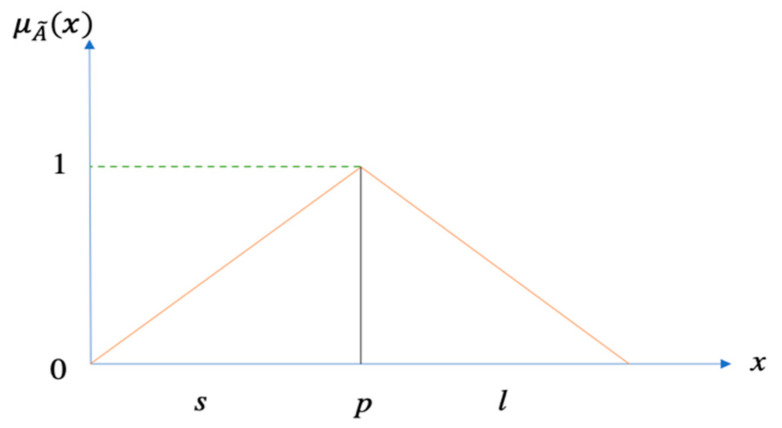
The membership functions of triangular fuzzy numbers. Reprinted with permission from Ref. [64]. Copyright (2021), Elsevier Ltd.

As an extension to the standard AHP technique, Fuzzy AHP was utilized to assess the weight of decision elements due to the increasing significance of the fuzzy set principle in the scope of multicriteria approaches. The key intent of this act was to perform a comparative study of two arrangements of weights and also to define the effect of the fuzzy scale on component weights and, consequently, on decision levels [61]. In complex situations, drivers generally fail to define their priorities because of the vague characteristics of complex problems [17]. To mitigate the ambiguity of individual thinking and handle uncertainty problems, [62] first created the fuzzy set theory, which focused on the rationality of ambiguity. As the human point of view was utilized in weighting the most important factors influencing the studied complex problem,

$\tilde{A}=\left(s,\;p,\;l\right)$, where the parameters *s*, *p*, and *l* signify the smallest viable value, the most likely value, and the leading potential value, respectively, which explain a fuzzy case [63] is presented in Equation (4). A triangular membership function of $\tilde{A}$ is presented in Figure 2.

(4) $$\mu_{\tilde{A}}\left(x\right)=\left\{\begin{array}{ll} \frac{x-s}{p-s}, & s\leq x\leq p\\ \frac{l-x}{l-p}, & p\leq x\leq l\\ 0, & otherwise \end{array}\right.$$

The main operation steps that can be conducted on triangular fuzzy numbers [62] are the following.

Addition:

(5) $$\left(s_{1}+s_{2},p_{1}+p_{2},\;l_{1}+l_{2}\right)$$

Multiplication:

(6) $$\left(s_{1}\times s_{2},\;p_{1}\times p,\;l_{1}\times l_{2}\right)$$

Subtraction:

(7) $$s_{1}-l,\;p_{1}-p_{2},\;l_{1}-s_{2})$$

Division:

(8) $$\left(\frac{s_{1}}{l_{2}},\frac{p_{1}}{,p_{2}},\frac{l_{1}}{s_{2}}\right)$$

Reciprocal:

(9) $${\tilde{A}}^{-1}=\left(\frac{1}{s_{1}},\frac{1}{p_{1}},\frac{1}{l_{1}}\right)$$

In this study, the calculation approach is based on fuzzy numbers for which their values are presented in Table 6.

### 2.5. The Proposed Fuzzy AHP

**Figure 3:**
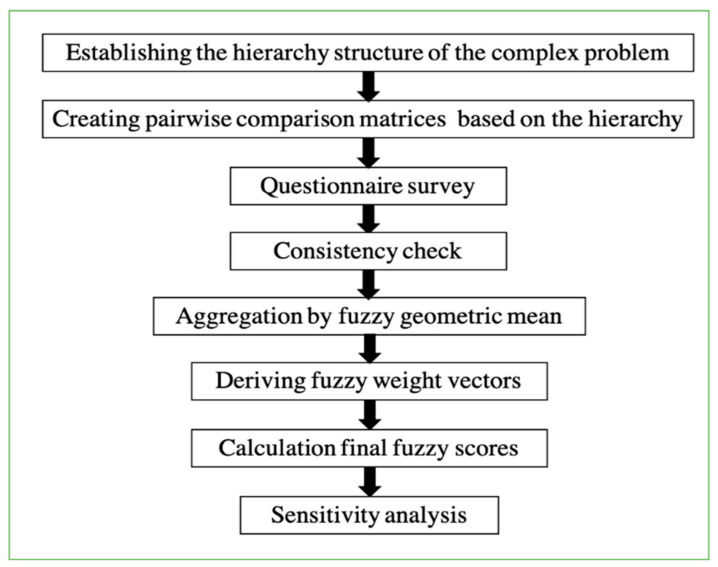
The main step of conducting AHP in Fuzzy light conditions.

Zadeh et al. [62] presented a model of linguistic variables to convey individual discernment in terms of fuzzy arrangements as a substitute of crisp values. Van et al. [66] utilized the first fuzzy extension of the AHP technique to develop Saaty’s AHP procedure with triangular fuzzy numbers. Chang et al. [67] used triangular fuzzy numbers to assess the weight vectors under the individual measure based on pairwise comparisons. This study utilized an integrated fuzzy AHP model to measure the effect of critical factors on lane changing in a two-level hierarchical lane change structure. Figure 3 represents the flowchart for the main steps of the conducted model. Furthermore, after the defuzzification step, measuring the consistency of all pairwise comparison matrices is an important step, and it is adopted by computing the values of the consistency index (CI) and consistency ratio (CR) by applying Equations (2) and (3), respectively.

The fuzzy AHP method is observed as an appropriate approach for this research study, and the following steps were conducted:

Step#01: Establishing the hierarchy structure of the complex problem;
Step#02: Designing the pairwise comparison matrices by considering all factors in a hierarchy structure;
Step#03: Evaluating pairwise comparison matrices by utilizing the fuzzy number scale (Table 6);
Step#04: Aggregating the preferences of all evaluators by using geometric mean;
Step#05: Calculate the final overall weights for all factors (Table 7);
Step#06: Weights’ defuzzification;
Step#07: Calculating th absolute weight scores by considering branch relations in the case of multilevel;
Step#08: Checking consistency;
Step#09: Sensitivity analysis.

The fuzzified weights in proposed model are conducted by using the following:

(10) $${\tilde{g}}_{i}={\left({\tilde{a}}_{i1}⊗{\tilde{a}}_{i2}⊗{\tilde{a}}_{i3}⊗{\tilde{a}}_{i4}⊗{\tilde{a}}_{i5}\right)}^{1/n}$$

(11) $${\tilde{w}}_{i}={\tilde{g}}_{i}\;{\left[{\tilde{g}}_{1}⊗{\tilde{g}}_{2}⊗{\tilde{g}}_{3}⊗{\tilde{g}}_{4}⊗{\tilde{g}}_{5}\right]}^{-1}$$

where ${\tilde{a}}_{ij}$ is fuzzy preference value of dimension *i* to factor *j*; thus, ${\tilde{g}}_{i}$ is the geometric mean of fuzzy preference value of factor *i* to each factor, and ${\tilde{w}}_{i}$ includes the fuzzy weights of the *i*-th factors and can be adopted by a triangle fuzzy number, defined by ${\tilde{w}}_{i}=\left(sw_{i},\;pw_{i},\;lw_{i}\right)$ $sw_{i},\;pw_{i},\;and\;lw_{i}$, considered for the upper, middle, and lower numbers of the fuzzy weight of the *i*-th dimension.

As the last action, sensitivity analysis is employed. In our case, we increased the weight of individual criteria, and the changes within the priorities were stable. This enables an understanding of the effects of changes in the ranking of main criteria and sub-criteria and helps the decision-maker to check for robustness throughout the process. In general, sensitivity analysis can be adopted by increasing or decreasing the weight of individual criteria, resulting in changes within the priorities; thus, the ranking of the alternatives is often observed. Sensitivity analysis, therefore, provides information on the stableness of the ranking. If ranking is extremely sensitive to small changes within the standard weights, a careful review of the weights is usually recommended. In addition, decision criteria should be included as a sensitive ranking point to a weak discrimination potential for this set of criteria.

## 3. Results

The final measured weights based on the FAHP model are very reliable as it provides more consistent outcomes compared to the standard AHP method. A previous study noticed that the AHP method generates and deals with a highly unbalanced scale of judgment, and it does not study the uncertainty connected with the mapping of human judgment to a number by natural linguistics [68]. The final overall weights for all factors were measured based on a fuzzy AHP model, as shown in Table 7. For level 1, the weight score was observed to be high for the factor ‘Traffic characteristics’ (0.5404) followed by ‘Human’ (0.2232). While the weight score was estimated to be the lowest for factor ‘Light conditions’ (0.0882) as compared to other specified factors. For level 2, the weight score was observed to be high for factor ‘Daytime light’ (0.8193) followed by ‘Road type’ (0.6215), while the weight score was estimated to be the lowest for the factor ‘Illiteracy’ (0.1084). 

While the ranks for the main factors in the first level of the lane change model were estimated based on FAHP measured weights, as presented in Table 8, the results revealed ‘Traffic Characteristics’ as the highest rank factor because it has the biggest weight score (0.5404) followed by ‘Human’ factor where its weight score is (0.2232). From the findings of the previous study, the effect of specified traffic characteristics on lane changing was observed highly significant [69]. Furthermore, FAHP results estimated ‘Light Conditions’ as the least rank factor with a weight score (0.0882) followed by ‘Road Characteristics’ with a weight score of (0.1847). The lowest environmental effect was observed in connection with enhanced traffic safety and high traffic competence [70].

For level 2, the FAHP model results showed ‘Traffic Volume’ as the most significant factor with a weight score (0.22) followed by ‘Speed’ factor with weight score (0.1734) as compared to other observed factors. It was observed that traffic volume has a major influence on the overtaking frequency and following gap, which are naturally related to lane changing [71,72], while a recent study estimated that different types of speed significantly influence lane changing, such as speed distribution, average speed variation [14], and speed above speed limit [16]. Furthermore, based on final weights, the model outcomes assessed ‘Night Light’ as the least critical factor with weight scorer (0.0159) followed by the ‘Illiteracy’ factor with a weight score of (0.0242), while the ranks of other factors based on weight scores are depicted in Table 9.

### 3.1. Sensitivity Analysis

In model development utilizing AHP, sensitivity analysis is a significant procedure in deciding if the outcome is robust and implementable. It is an important process for better forecasting; accordingly, future planning will have more accuracy [33]. The sensitivity analysis process is one of the AHP steps in order to apply and execute the AHP approach. In this study, sensitivity analysis has been conducted as follows: In the first level, the ‘traffic characteristics’ factor weight was amended from 0.5404 to 0.5500. The slight change in the factor weight from 0.5404 to 0.55 is for detecting the stability of the other factor’s weight, which is the aim of conducting sensitivity analysis. The weight scores of the other factors in this level were modified in order to maintain a score of one for the total calculation of weight scores. 

Correspondingly, the score of lower-level factors was altered (as Equation (7) specifies). In sensitivity assessment, the first ranking level was held, and the variation in ranking was analyzed based on previous and current traffic safety assignments in a city. This proposition might reason an adjustment in the significance of evaluators. As shown in Table 10, the applied modification has not changed the factor rank or order.

Changes have been slightly detected in the second level. As shown in Table 11, the sensitivity of the sample test is small; for example, the ‘traffic composition’ factor has been changed slightly from the eight position to the seventh position, while the factor ‘following distance’ is now in the fourth position instead of the fifth position. The other three factors have not changed.

### 3.2. Discussion

The use of fuzzy AHP is considered indispensable when inaccuracy in decision-making must be removed. To determine the applicability of the designed model, a real-life traffic safety problem (frequent lane changing) is designated, ranking the highly critical factors influencing lane changing based on the responses of drivers. Based on subjective decisions, uncertainty in the evaluation procedure is incorporated by using FAHP. Applying AHP in fuzzy light conditions has been proven to be successful based on the results since some drivers are not fully attentive of the significant parts in pairwise comparisons, thus allowing more flexible numbers aided in the acquisition of a highly reliable ranking. The objectivity of the model was supported by sensitivity analyses that examine a range for the weights’ values and corresponding to alternative scenarios. The fuzzy method can be proposed for all decision support fields in which layman evaluators assess the elements of the decision system, mainly in techniques in which pairwise comparisons are applied. In addition, the proposed model enables experts to be familiarized with the complete assessment process. This significance has been verified by our survey data. The projected integrated method could aid decision-makers in concentrating on high-ranked critical factors influencing frequent lane changes to increase the safety of roads. A study utilized the AHP model [73] for apps mapping and expert opinions to control risky driving behavior, which may contribute to road safety. Previous study findings provided safety direction for the safe lane changing of vehicles in the system of the Internet of Vehicles, which decreases the incidence of road traffic collisions and safeguards sustainability and traffic operation safety in an effective manner [74]. A recent study proposed a model that can explore significant developments in safe lane-changing with respect to connected and autonomous vehicles (CAVs) in heterogeneous traffic flow for both connected and autonomous and human-driven vehicles (HVs) in the future [75]. In addition, traffic safety campaigns and drivers training based mainly on the driver’s compliance with speed limits, lane-changing rules, and use of intelligent traffic systems should be held in educational institutes to address considerations, which may result in improved perceived risk and lower engagement in risky driving behaviors [76,77,78,79].

## 4. Conclusions

In this work, the effect of critical factors and subfactors on lane-changing was analyzed for traffic safety systematically in the fuzzy system. The framework of our study has been adopted after numerous iterations aimed to outline relevant key factors. The evaluation results of this study highlighted the most critical factors that could influence frequent lane change behavior and further cause negative effects on road safety. For level 1, the ‘traffic characteristics’ factor was detected as the most critical factor, while the second most critical factor was ‘human’, as followed by ‘road characteristics’. For level 2, the FAHP model results assessed ‘traffic volume’ as the most significant factor, followed by the ‘speed’ factor. The evaluation of significant traffic parameters instigating frequent lane changes could increase our consideration of lane changing risk and would encourage the improvement of a more sustainable transport network. Some significantly estimated factors related to ‘Traffic characteristics’ (speed) and ‘Road characteristics’ (road type) should be considered for the development of improved road design for sustainable road safety. These essential evaluations could be valuable for drivers with respect to being aware of their individual traffic risks for a specified region. Linkage of the assessed data with traffic authorities may help to implement effective local road safety strategies. Traffic safety campaigns based primarily on the driver’s compliance with lane-changing rules should be held in public and private sector institutes to address the attention, which may result in improved perceived risk when possible issues arise in performing a decision concerning the risk mechanisms related to frequent lane changing by utilizing a single form of fuzzy linguistic expressions. Consequently, the individual can use the performance of numerous forms of fuzzy sets theory for determining other related problems of road safety and for advanced vehicle automation subjects. Moreover, more test data from different concerned regions should be used to check the validity of the algorithm that has been considered in this research in follow-up research. Comparing different data sets will enable us to develop this work in improving proactive behavioral systems.

## Figures and Tables

**Table 1:** Summary of Fuzzy AHP applications in road transport-related studies.

Authors (Year of Publication)	Applications
Srisawat et al., 2017 [23]	Estimate the quality of transport logistics on a regional scale
Nanda and Singh, 2018 [24]	Evaluate the factors of road incidents
Danish Farooq and Sarbast Moslem, 2019 [25]	Estimated the significant driver behavior factors affecting the highway safety in the context of the city of Budapest, Hungary
M. Gul et al., 2018 [26]	The authors presented a risk assessment model based on FAHP for means in hazardous substance transportation
Shalini Kanuganti et al., 2016 [27]	Investigate the ranking of safety essentials of a particular group of rural roads
Pandian et al., 2016 [28]	Presented a model to optimize/minimize blind areas/spots for heavy transport vehicles
Yaqin He and Shengpin Du, 2016 [29]	A quantitative model of emergency categorization offered by focusing traffic guarantee power during the collision

**Table 2:** Descriptive statistics of study participants.

Variable Description	Frequency	Percentage (%)
Number (N)	70	100
*Age (years)*		
18–30	14	20
31–50	34	48.5
51 above	22	31.5
*Gender*		
male	63	90
female	07	10
*Duration of driving license* (years)		
1–5	11	15.71
6–15	37	52.85
16–25	22	31.42
*Education*		
Bachelor’s degree	33	47.14
MSC/PhD	37	52.86

**Table 3:** An Example of Questionnaire survey for level 1.

Comparing the Selected Factors Importance in Response to Rrequent Lane-Changing				
	Traffic Characteristics	Human Attributes	Road Characteristics	Light Conditions
Traffic characteristics	(1, 1, 1)	(2, 3, 4)	(4, 5, 6)	(6, 7, 8)
Human	(1/2, 1/3, 1/4)	(1, 1, 1)	(6, 7, 8)	(4, 5, 6)
Road characteristics	(1/4, 1/5, 1/6)	(1/6, 1/7, 1/8)	(1, 1, 1)	(6, 7, 8)
Light conditions	(1/6, 1/7, 1/8)	(1/4, 1/5, 1/6)	(1/6, 1/7, 1/8)	(1, 1, 1)

**Table 4:** Importance of specified factors in related traffic safety studies.

Main Factor	Sub-Factor	Explanation and Related Reference
Traffic Characteristics (F1)	Traffic Volume (F1.1)	Traffic volumes were identified as the highly significant factors for modeling the driving behavior [40]
	Traffic Composition (F1.2)	Traffic composition has statistically major impacts on collision occurrence [41]
	Following Distance (F1.3)	To ensure the safety distance for lane changing, a safe car-following distance should be reserved [42].
	Speed (F1.4)	High-speeds variations within the same lane characterize situations with decline levels of service (LOS) and consequently unstable flow. These situations can cause greater accident risk [43]
	Vehicle Type (F1.5)	Vehicle type has been utilized in numerous traffic crash studies [44,45]
Human (F2)	Carelessness (F2.1)	Previous studies confirmed that drivers with careless driver behavior might considerably raise the risk of traffic collisions [46,47]
	Illiteracy (F2.2)	The study results revealed that most casualties in traffic collisions were illiterates for different age groups [48]
	Violation of Traffic Rules (F2.3)	Traffic violations were noted to be the leading risks threatening road safety [49]
	Training (F2.4)	Driving behavior is affected by training, experience, and personal characteristics [50]
Road Characteristics (F3)	Road Type (F3.1)	A previous study analyzed the relationship between type of road infrastructure and crash involvement [51]
	Road Surface (F3.2)	Previous study analysis indicated that deformations on pavement surface have a positive impact on lane-changing [52]
	Grade (F3.3)	Road safety problems may appear due to upgrade or downgrade sections [51]
Light conditions (F4)	Daytime light (F4.1)	Dark lighting conditions are more likely to lead to fatal or severe injury crashes compared with daylight [53,54,55]

**Table 5:** Random Index values based on matrix size.

*n*	*RI*
1	0
2	0
3	0.58
4	0.9
5	1.12
6	1.24
7	1.32
8	1.41

**Table 6:** Membership function of linguistic scale [65].

Linguistic	Scale of Fuzzy Number
Extremely important	(8, 9, 10)
Very strong important	(6, 7, 8)
Important	(4, 5, 6)
Moderately important	(2, 3, 4)
Equally important	(1, 1, 1)
Intermediate values	(7, 8, 9), (5, 6, 7), (3, 4, 5), (1, 2, 3)

**Table 7:** Factor weight scores affecting frequent lane changing based on expert drivers’ responses based on the Fuzzy AHP model.

Level 1		Level 2	
Main Factor	Weight	Sub-Factor	Weight
Traffic Characteristics	0.5404	Traffic Volume	0.4071
		Traffic Composition	0.1285
		Following Distance	0.1565
		Speed	0.3209
		Vehicle Type	0.1150
Human	0.2232	Carelessness	0.2585
		Illiteracy	0.1084
		Violation of Rules	0.3989
		Training	0.2748
Road Characteristics	0.1847	Road Type	0.6215
		Road Surface	0.2280
		Grade	0.1904
Light conditions	0.0882	Daytime light	0.8193
		Night Light	0.1807

**Table 8:** The final weight scores for the main factors in the first level.

Factor	Weight	Rank
Traffic Characteristics	0.5404	1
Human	0.2232	2
Road Characteristics	0.1847	3
Light conditions	0.0882	4

**Table 9:** The final weight scores for subfactors in the second level.

Factor	Local Weight	Final Weight	Rank
Traffic Volume	0.4071	0.2200	1
Traffic Composition	0.1285	0.0694	8
Following Distance	0.1565	0.0846	5
Speed	0.3209	0.1734	2
Vehicle Type	0.1408	0.0761	6
Carelessness	0.2585	0.0577	10
Illiteracy	0.1084	0.0242	13
Violation of Rules	0.3989	0.0890	4
Training	0.2748	0.0614	9
Road Type	0.6215	0.1148	3
Road Surface	0.2280	0.0421	11
Grade	0.1904	0.0352	12
Daytime light	0.8193	0.0723	7
Night Light	0.1807	0.0159	14

**Table 10:** The final weight scores for main factors in the first level after sensitivity analysis.

Factor	Weight	Weight after the Sensitivity Analysis	Rank
Traffic Characteristics	0.5404	0.5500	1
Human	0.2232	0.2100	2
Road Characteristics	0.1847	0.1700	3
Light conditions	0.0882	0.0700	4

**Table 11:** The final weight scores for main factors in the second level after sensitivity analysis.

Factor	Local Weight	Final Weight	New Rank
Traffic Volume	0.4071	0.223905	1
Traffic Composition	0.1285	0.070675	7
Following Distance	0.1565	0.086075	4
Speed	0.3209	0.176495	2
Vehicle Type	0.1408	0.07744	6
Carelessness	0.2585	0.054285	10
Illiteracy	0.1084	0.022764	13
Violation of Rules	0.3989	0.083769	5
Training	0.2748	0.057708	8
Road Type	0.6215	0.105655	3
Road Surface	0.2280	0.03876	11
Grade	0.1904	0.032368	12
Daytime light	0.8193	0.057351	9
Night Light	0.1807	0.012649	14

## Data Availability

All accompanying data are provided in the manuscript.
